# neomerDB: a comprehensive database of neomer biomarkers in cancer

**DOI:** 10.1093/database/baag006

**Published:** 2026-02-12

**Authors:** Kimonas Provatas, Candace S Y Chan, Ioannis Kerasiotis, Eleftherios Bochalis, Akshatha Nayak, Brad E Zacharia, Georgios A Pavlopoulos, Wei Li, Ilias Georgakopoulos-Soares

**Affiliations:** Division of Pharmacology and Toxicology, College of Pharmacy, The University of Texas at Austin, Dell Pediatric Research Institute, 1400 Barbara Jordan Boulevard, Austin, TX 78723, United States; Institute for Personalized Medicine, Department of Biochemistry and Molecular Biology, The Pennsylvania State University College of Medicine, 500 University Drive, Hershey, PA 17033, United States; Division of Pharmacology and Toxicology, College of Pharmacy, The University of Texas at Austin, Dell Pediatric Research Institute, 1400 Barbara Jordan Boulevard, Austin, TX 78723, United States; Institute for Personalized Medicine, Department of Biochemistry and Molecular Biology, The Pennsylvania State University College of Medicine, 500 University Drive, Hershey, PA 17033, United States; Division of Pharmacology and Toxicology, College of Pharmacy, The University of Texas at Austin, Dell Pediatric Research Institute, 1400 Barbara Jordan Boulevard, Austin, TX 78723, United States; Institute for Personalized Medicine, Department of Biochemistry and Molecular Biology, The Pennsylvania State University College of Medicine, 500 University Drive, Hershey, PA 17033, United States; Division of Pharmacology and Toxicology, College of Pharmacy, The University of Texas at Austin, Dell Pediatric Research Institute, 1400 Barbara Jordan Boulevard, Austin, TX 78723, United States; Institute for Personalized Medicine, Department of Biochemistry and Molecular Biology, The Pennsylvania State University College of Medicine, 500 University Drive, Hershey, PA 17033, United States; Division of Pharmacology and Toxicology, College of Pharmacy, The University of Texas at Austin, Dell Pediatric Research Institute, 1400 Barbara Jordan Boulevard, Austin, TX 78723, United States; Institute for Personalized Medicine, Department of Biochemistry and Molecular Biology, The Pennsylvania State University College of Medicine, 500 University Drive, Hershey, PA 17033, United States; Department of Neurosurgery, Penn State Milton S. Hershey Medical Center, 500 University Drive, Hershey, PA 17033, United States; Institute for Fundamental Biomedical Research, BSRC “Alexander Fleming”, 34 Fleming Street, 16672, Vari, Athens, Greece; Department of Computational Biology, Mohamed bin Zayed University of Artificial Intelligence (MBZUAI), Masdar City, Abu Dhabi, United Arab Emirates; Division of Hematology and Oncology, Department of Pediatrics, Penn State College of Medicine, 500 University Drive, Hershey, PA 17033, United States; Division of Pharmacology and Toxicology, College of Pharmacy, The University of Texas at Austin, Dell Pediatric Research Institute, 1400 Barbara Jordan Boulevard, Austin, TX 78723, United States; Institute for Personalized Medicine, Department of Biochemistry and Molecular Biology, The Pennsylvania State University College of Medicine, 500 University Drive, Hershey, PA 17033, United States

## Abstract

The development of biomarkers for population screening, early cancer detection, monitoring, and recurrence surveillance offers substantial potential to improve patient outcomes and save lives. Nullomers are short *k*-mers that are absent from a human genome, and neomers are the subset of nullomers that emerge recurrently due to somatic mutations during cancer development. Here, we have developed neomerDB, a database that encompasses a catalogue of neomers across cancer types and organs. We examined 10 000 whole exome sequencing and 2658 whole genome sequencing tumour-matched samples and identified the set of neomers associated with each cancer type and organ. We also analysed 76 215 whole genomes and 730 947 whole exomes of individuals from diverse ancestries, from which we removed nullomers and neomers that can arise due to germline variants in the population. Finally, we conducted a case study demonstrating that neomers can be utilized to detect glioblastoma from liquid biopsy samples (*n* = 38), utilizing cell-free DNA and cell-free RNA, achieving a Receiver Operating Characteristic - Area Under the Curve score of 0.98 and a precision-recall score of 0.99. neomerDB is a user-friendly database that enables advanced searches, provides interactive visualizations, and download options for neomer biomarkers. neomerDB is publicly available at https://neomerDB.com/.

## Introduction

Cancer is the second leading cause of death, and it is estimated that 40% of the population will be diagnosed with cancer in their lifetime [1]. Detection of cancer at the earliest stage can lead to timely intervention and improved clinical outcomes [2]. Nevertheless, tumours are often detected at a symptomatic, advanced stage, at which treatment success rates decline precipitously. In recent years, liquid biopsies have emerged as a promising method for cancer detection [3]. As cells in the body die, they release DNA and RNA in the bloodstream, known as cell-free DNA/RNA [4]. By analysing cell-free DNA/RNA, it is possible to detect cancer-associated mutations, methylation patterns, fragmentomic signatures, and other molecular features indicative of tumour presence [5, 6]. However, liquid biopsy-based testing can be technically challenging since the amount of tumour DNA and RNA present in the blood and other bodily fluids is tiny, particularly at early stages of the disease, necessitating sensitive biomarkers.

Nullomers are short *k*-mer sequences absent from a genome [7]. We have previously shown that a subset of nullomers recurrently emerges in tumour samples, which we termed neomers [8]. We have developed a novel approach for detecting cancer from liquid biopsies using neomers. By analysing thousands of cancer genomes, we identify these short DNA and RNA sequences that are absent from the healthy genome but recur in tumour samples. We have demonstrated that a unique set of neomers is associated with each cancer type [8, 9]. We and others have analysed liquid biopsy-derived cell-free DNA and cell-free RNA from healthy controls and patients diagnosed with different cancer types, including breast, lung, colorectal, gastric, liver, oesophageal, stomach, and ovarian cancers, showcasing the ability of nullomers and neomers to detect cancer [8–14]. We have also optimized our approach further by removing nullomers, neomers, and related sequences, which could emerge in the human population due to germline variants [8, 15]. Thus, we can generate curated, cancer-type-specific neomer panels that serve as sensitive and specific biomarkers. Nevertheless, a resource that provides neomers and related sequences across cancer types, which could enable their wider adoption, is currently lacking.

**Figure 1:**
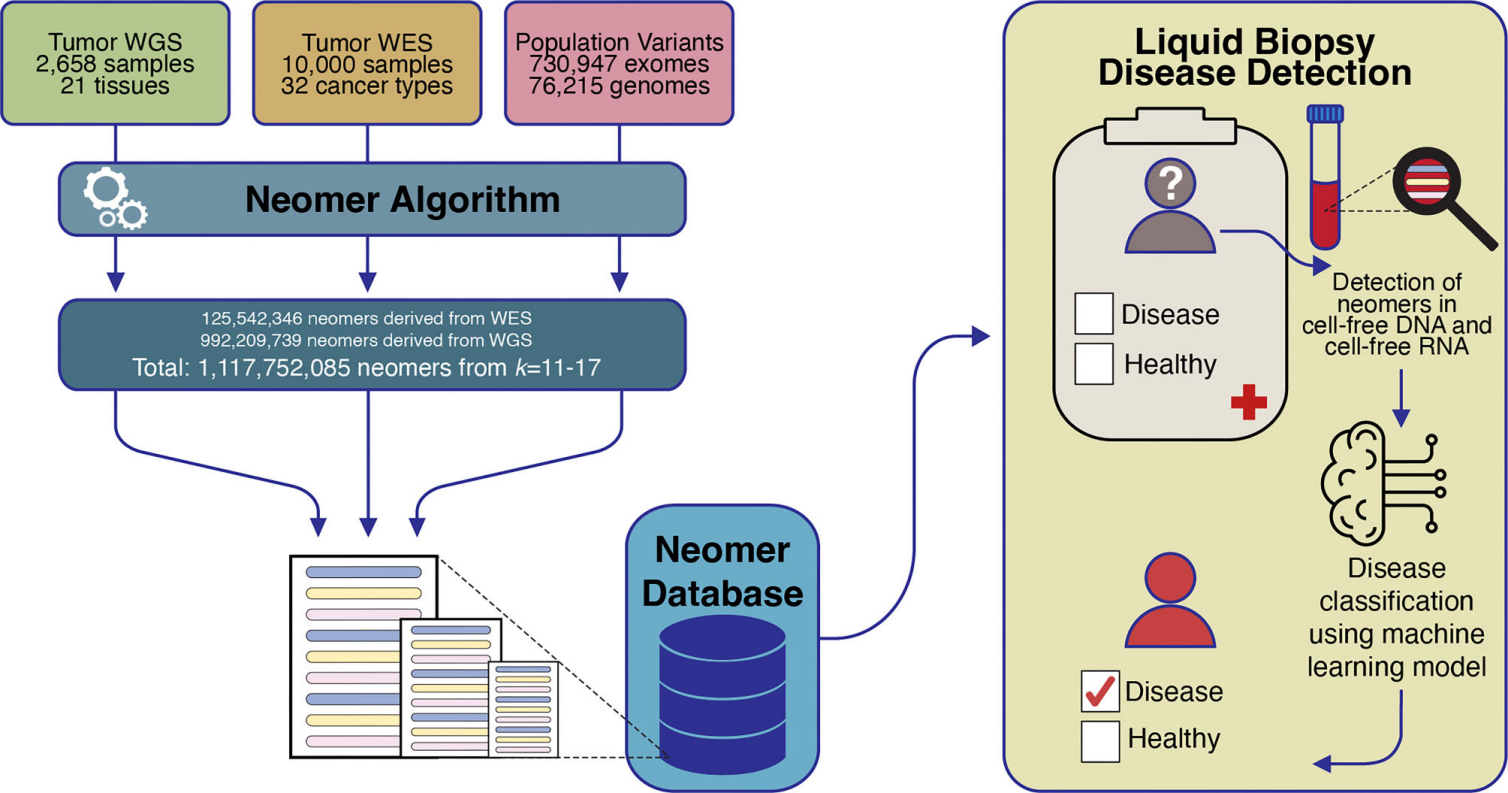
Overview of neomerDB. The database integrates neomers derived from two different approaches: (1) 2658 whole genome sequencing tumour-normal paired samples and (2) 10 000 whole exome sequencing tumour-normal paired samples. Neomers identified from germline variants, from 76 215 whole genomes and 730 947 whole exomes, are filtered with thresholds adjustable by the user. The neomer profiles are used for cancer biomarkers in liquid biopsies through their incorporation in machine learning classification models.

Here, we present neomerDB, the first dedicated database for neomer biomarkers. Utilizing data from 2658 whole-genome and 10 000 whole-exome sequenced tumour samples along with matched controls, we identified neomers of varying lengths (11–17 bp). We also analysed 76 215 germline whole genomes and 730 947 germline whole exomes from individuals of diverse ancestries, enabling the exclusion of nullomers and neomers that could result from germline variants that can be found in the human population. We provide interactive tables and visualizations that allow users to explore neomer data alongside patient metadata, and clinically relevant information (Fig. 1). The database offers advanced filtering options that take into account the probability of neomer occurrence due to germline variation, both in the general population and in specific subpopulations. Other filters include the selection of neomers detected across all cancer types or neomers detected in a specific cancer type or organ, filtering neomers by the stage of the tumour in which the neomers were found, and the recurrence threshold across cancer patients. Finally, we performed a proof-of-concept case study and showed that we can use neomers to detect glioblastoma patient samples using cell-free DNA and RNA from liquid biopsies (Receiver Operating Characteristic - Area Under the Curve (ROC-AUC) = 0.98; precision-recall = 0.99). Taken together, the database is user-friendly and provides a wealth of *k*-mer biomarkers for cancer research.

## Materials and methods

### Data collection

Somatic mutations, including single-nucleotide polymorphisms, doublet-base substitutions, and short indels, were derived for 2658 individuals across 21 tissues for whole genome sequenced tumour and matched control samples from [16] and 10 000 whole exome sequenced tumour and matched control samples spanning 32 cancer types from [17]. Clinical information, including age, gender, cancer type, tumour stage, and overall survival, was integrated into the database.

### Neomer extraction algorithm

The CHM13v2 reference assembly of the human genome was used to support the nullomer detection in the reference human genome. Nullomer extraction was also performed directly from the mutation files, in their reference human assembly, with algorithmic optimizations over our previously developed algorithms [8, 9], enabling scalable neomer identification for any *k*-mer length. The neomer extraction pipeline processes mutation data to identify candidate neomers. Initially, a KMC database file [18] is generated that holds all *k*-mers found in CHM13.v2 [18]. The core processing step is carried out by a Python-based command-line tool. Mutation data are read from the MAF file, and reference sequences are retrieved based on genomic coordinates using pyfaidx (v.0.8.14) [19], allowing for constant-time sequence access. For each mutation, a flanking window is generated that includes the mutated region plus an additional *k* + 30 bases on both the left and right sides. This is performed for both the reference and the mutated sequences. Once both sequences are constructed, the tool performs a set difference operation to identify *k*-mers present in the mutated sequence but absent from the reference sequence. These unique *k*-mers are considered potential neomers. Our pipeline treats each mutation as an independent event. While spatially proximal mutations could theoretically interact, the inherent complexity of haplotype phasing and the statistical rarity of such occurrences within short *k*-mer windows led us to adopt this standard analytical practice. Each potential neomer is then checked against the KMC database file [18]; if it is not found in the database, it is classified as a neomer and written to disk.

### Nullomers and population variants

Population variants were derived from gnomAD v4 [20] for 730 947 whole exomes and 76 215 whole genomes. Allele frequency (AF) was estimated across the population cohort and for the following ancestries: East Asian (EAS), African-American/African (AFR), Finnish (FIN), Amish (AMI), Latino/Admixed American (AMR), non-Finnish European (NFE), South Asian (SAS), and Ashkenazi Jewish (ASJ). Nullomers from common population variants were derived across all individuals and for all ancestries and have been integrated into neomerDB. Using the AF of each germline variant capable of generating a given nullomer or neomer, we estimated the probability of its occurrence under the assumption of conditional independence of germline mutations.

### Common variant extraction pipeline

The common variant extraction pipeline identifies *k*-mers that may arise from common variant mutations, following a defined sequence of processing steps. The input variant dataset was derived from gnomAD v4 [20] for reference assembly GRCh38, and filtered to retain only those entries where at least one population-specific variant probability exceeded 5%. As in the neomer extraction pipeline, initially, a KMC database file [18] is generated that holds all *k*-mers. For each common variant, the reference sequence is retrieved using pyfaidx (v.0.8.14) [19], enabling constant-time access to genomic regions. A flanking window of *k* + 30 nucleotides is generated on both sides of the variant for both the reference and the mutated sequences. A set difference operation is performed between the mutated and reference *k*-mers to identify candidate nullomers. Similar to the neomer extraction methodology, each common variant is processed independently. This approach aligns with common practice for large-scale variant analysis and addresses the vast majority of variant contexts, considering the low probability of multiple interacting common variants within the *k*-mer definition. Each resulting nullomer is associated with probability data from gnomAD, including the overall probability of being a common variant and per-subpopulation probabilities. To estimate the likelihood that a given *k*-mer is not a product of common variation, the pipeline computes the complement of the variant probability for each associated mutation and multiplies these values across all contributing variants. This yields a composite probability that the *k*-mer does not arise from any common variant. The final output consists of *k*-mers that are nullomers, absent from the reference genome but generated by common variants, along with their corresponding probabilities across all populations and subpopulations, written to disk for database integration. These probabilities then act as a confidence level for each neomer in the database and can be tuned for different populations to remove neomers that could be attributed to common variant mutations.

### Web application architecture

neomerDB is a full-stack web-based application designed for efficient exploration and analysis of cancer signatures. The backend is implemented in Go (1.23.2) using the lightweight and high-performance Gin framework (1.10). For analytical processing and high-speed querying of large datasets, neomerDB integrates DuckDB via the go-duckdb (v1.8.3) driver, enabling in-process OLAP capabilities optimized for analytical workloads. The frontend is built using React (v19.1.0), offering a responsive and interactive user experience. User interface (UI) development incorporates Material UI (v7.1.0) for consistent, accessible, and component-rich design. Data visualization is powered by D3 (v7.9.0) and Apache ECharts (v5.6.0), integrated via echarts-for-react (v3.0.2), providing rich and customizable charting capabilities. Application state management, routing, and tabular data handling are efficiently managed through TanStack, including TanStack Query (v5.76.1), TanStack Router (v1.120.5), and TanStack Table (v8.21.3) [MIT License]. The application is built using Vite (v6.3.5) [MIT License] and features Swiper (v11.2.8) [MIT License] for modern touch-enabled sliders. Type safety and maintainability are ensured through TypeScript (v5.8.3) [Apache License 2.0]. This architecture ensures a responsive, high-performance platform suitable for real-time cancer-specific data exploration and visualization.

### cfDNA and cfRNA extraction protocols

Qiagen’s miRNeasy Serum/Plasma Kit was used for the purification of cell-free total RNA from 200 μl of plasma. Manufacturer’s directions, including DNAase treatment, for the appropriate volume of starting sample were followed. RNA was eluted in 14 μl of nuclease-free water. Quantity and quality of the extracted RNA were checked using BioAnalyzer (Agilent Technologies) RNA 6000 pico Kit. Zymo’s MagicBead cfDNA isolation kit was used to extract cfDNA from 500 μl of plasma samples. Manufacturer’s instructions were followed, and resulting cfDNA was quantified using the BioAnalyzer High Sensitivity DNA Kit (Agilent Technologies).

### cfDNA and cfRNA library prep

Library from cfRNA was prepared for sequencing using SMARTer Stranded Total RNA-Seq Kit v3-Pico Input Mammalian Library Prep (Takara). Briefly, 350 pg of cfRNA, as calculated from the Bioanalyzer run, was used to prepare strand-specific and ribosomal RNA-depleted libraries with eight nucleotide unique molecular identifier (UMI) added through the reverse-transcription step and dual indices (Takara) during adaptor ligation following the manufacturer’s protocol. Final libraries were assessed for size distribution and concentration using the BioAnalyzer High Sensitivity DNA Kit (Agilent Technologies). Library from cfDNA was prepared for sequencing using the SRSLY Pico Plus Library Prep Kit (Claret Biosciences). The protocol efficiently creates sequencing molecules from both dsDNA and ssDNA. 2 ng of DNA was used and libraries prepared following the manufacturer’s protocol for without Enzymatic Shearing Module, following the steps for mono and di nucleosome peaks, bead purification option for moderate fragment retention, and with the addition of UMIs during primer extension and indexing PCR with premixed i5/Ui7 index primers (Claret). Final libraries were assessed for size distribution and concentration using BioAnalyzer High Sensitivity DNA Kit (Agilent Technologies).

### Sequencing

Libraries were prepared, pooled, and sequenced on NovaSeq 6000 (Illumina) to get paired-end 150 bp reads, according to the manufacturer’s instructions. Samples were demultiplexed using the bclconvert software (Illumina). Adaptors were not trimmed during demultiplexing. For cfRNA, Read 1 corresponds to the antisense sequence of the input RNA, while Read 2 corresponds to the sense strand. 8 bp UMIs + 3 bp UMI linker + 3 bp from Pico v3 SMART UMI Adapter from Read2 are trimmed before mapping. For cfDNA libraries, UMIs were extracted from the Index 1 reads, which were saved as FASTQ files. Each Index 1 read contains an 8 bp index sequence followed by a 9 bp UMI.

### Cell-free DNA and RNA analysis for neomer detection

Raw RNA-sequencing reads were deduplicated and filtered for UniVec, ERCC spike-in, and ribosomal sequences as previously described [9]. Neomer occurrences were counted using Jellyfish (version 2.2.10) [21]. Neomer counts were normalized using the counts per million method (as described in edgeR [22], implemented with the Python package conorm version 1.2.0). The stacked ensemble model performance was assessed using five-fold cross-validation with stratified sampling to preserve the original class distribution. Using the training fold, consistently expressed neomers within the control sample were filtered out. Additionally, features where more than 40% of all control samples exceeded a count threshold of 20 for RNA and 500 for DNA were removed. To address the class imbalance of the dataset, we used the SMOTE algorithm (as implemented in imbalanced-learn, version 0.12.4) to sample the controls. Scikit-learn (version 1.3.1) was used for the following machine learning steps. Data were preprocessed using MaxAbsScaler normalization for the RNA dataset, and StandardScaler for DNA. An ensemble stacking approach was used for the two data types. Logistic regression with balanced class weighting and XGBoost classifier was used for the RNA dataset, while a random forest model and XGBoost classifier were used for the DNA dataset. The four base models were combined using a stacking classifier that used logistic regression to integrate predictions from the RNA and DNA models. Model performance was evaluated using ROC-AUC and precision-recall AUC.

## Results

### neomerDB data annotation

**Figure 2:**
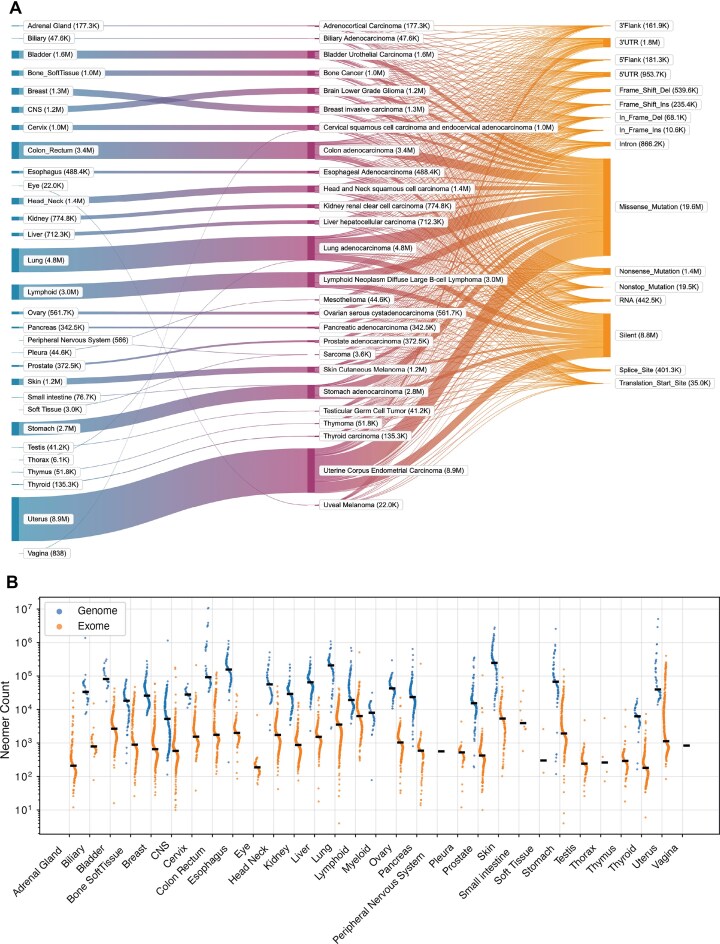
Statistics of neomerDB. (A) Sankey plot depicting the flow of exome neomers across 26 organs, 26 cancer types, and 16 variant classifications. Results are shown for neomers of 16 bp. In parentheses next to the tissue type, cancer type, and mutation category, the total number of nullomers detected is shown. (B) Neomer counts (16 bp) per patient across organs derived from genomes and exomes. The black line shows the median of neomer counts across patients.

The contents of neomerDB consist of exome neomers derived from 26 organs and 26 cancer types (Fig. 2A), and genome neomers derived from 21 organs and 34 cancer types (Supplementary Fig. 1). Each neomer entry is annotated with the mutation type from which the neomer was derived, revealing distinct patterns between genome and exome neomers. For exome neomers, we find that the majority of neomers arise from missense mutations, while the majority of neomers emerge from intergenic regions for genome neomers (Fig. 2A, Supplementary Fig. 1), consistent with previous findings [15]. The median number of neomers (16 bp) found in each patient is 857 for exome neomers and 33 474 for genome neomers (Fig. 2B). To evaluate the specificity of these sequences against common germline variation, we quantified the overlap between neomers and common variants across all libraries (lengths 11–17 bp). We defined a neomer as being ‘associated with a common mutation’ if its reported AF was strictly less than 1.0 in any annotated global or sub-population (including East Asian, African, and European cohorts), indicating the sequence exists as a known variant in the general population. We observed a sharp, length-dependent decay in this overlap (Supplementary Fig. 2). While shorter *k*-mers (11 bp) frequently coincided with common variants (∼76% for exomes and ∼77% for genomes), this association dropped significantly as length increased. At the standard 16 bp length, only 9.1% of exome neomers and 12.2% of genome neomers were associated with common variants, dropping further to 4.7% and 6.6%, respectively, at 17 bp. This demonstrates that longer neomer sequences are predominantly specific to somatic cancer events rather than common population polymorphisms.

### neomerDB database overview and functionality

**Figure 3:**
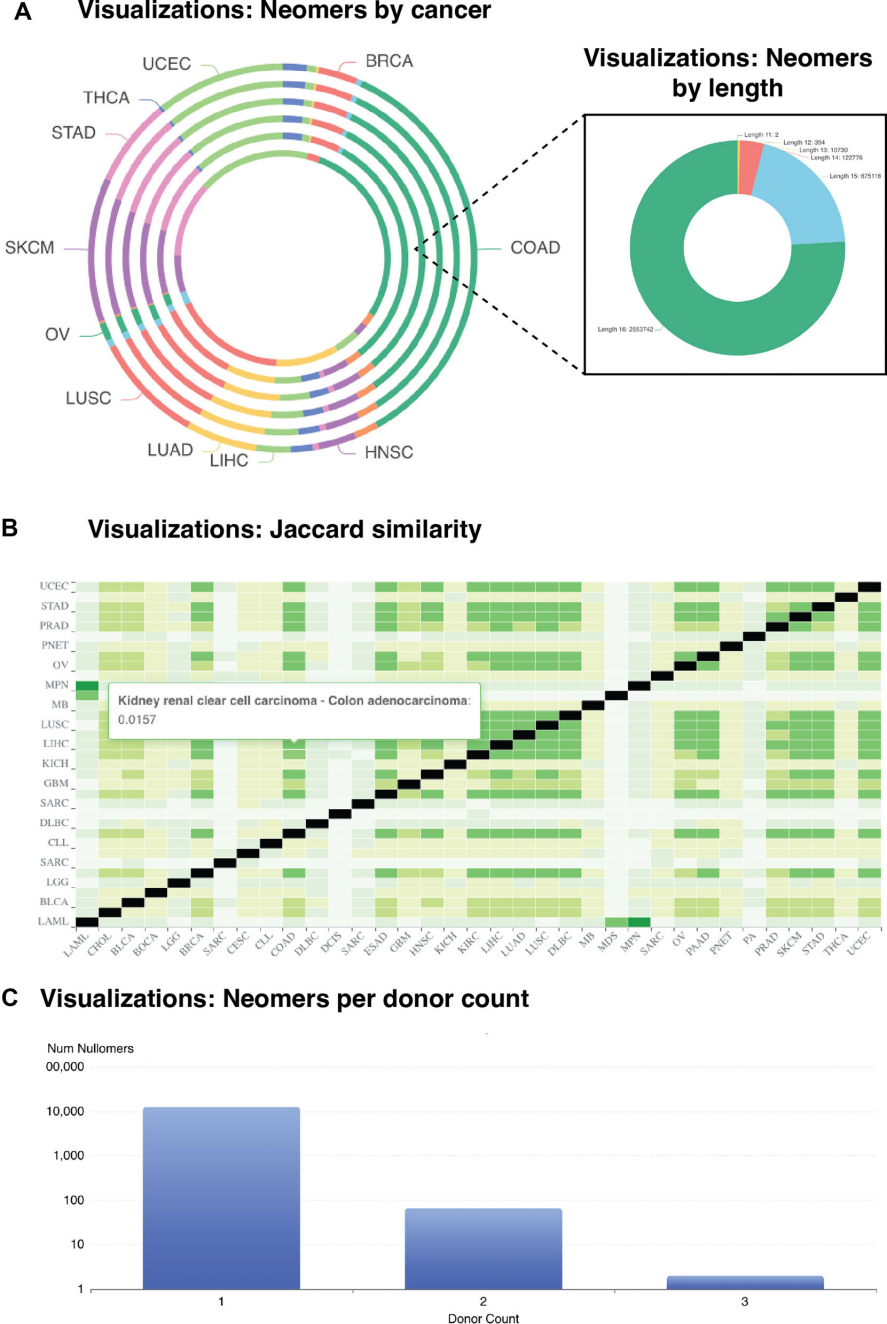
Dynamic, interactive tables displaying neomer profiles across genes and cancer types. (A) Conceptual overview of the neomerDB database interface. Coloured cursor symbols match the colour of their corresponding outlined view. (B–C) Interactive table and customizable query interfaces for (B) neomers and (C) patients. (D) View of neomer profiles for individual patients. (E) Neomer profile detailing the donors, organs, and cancers in which it has been identified.

Upon visiting neomerDB, users are presented with the homepage, which provides general information about the resource, including a summary of the associated publications with DOI references, and contact details. From the homepage, users can access various sections of the platform through the navigation bar. These sections include Patient Details (for both exome and genome data), the Neomers page (also separated into exomic and genomic views), Visualizations, and the Download section. The About, Help, and Privacy pages offer essential support and transparency to users of the neomerDB platform. The About page details the core components of the neomer extraction algorithm, the cfDNA/cfRNA analysis pipelines, and the underlying database architecture. The Help page introduces the concept of neomers and provides step-by-step guidance on using the platform’s features effectively (Fig. 3A–D). The Privacy page outlines the site’s data protection practices, security measures, and licensing terms, which adhere to the Creative Commons Attribution-NonCommercial-ShareAlike 4.0 (CC BY-NC-SA 4.0).

### Neomers (genomes/exomes)

Upon accessing the Neomers page, users are presented with a paginated display of all neomers corresponding to a selected length value (Fig. 3B). Both genomic and exomic datasets within this page maintain complete feature parity, ensuring consistent functionality and analysis capabilities across both dataset types. Users can refine the displayed neomers through various filtering criteria, including cancer-specific details, genomic regions, AF (serving as an indicator of confidence in identifying neomers as cancer signatures), and patient characteristics. Additionally, the interface provides functionality for users to customize and reorder column visibility, allowing tailored views according to specific analytical requirements. Users can subsequently download the filtered and customized datasets. Furthermore, the Neomers page includes analytical tools that facilitate statistical evaluation. These tools enable users to group neomers by available attributes, display the top-ranking groups in descending order, and analyse distribution patterns across various columns, supporting comprehensive assessment and interpretation of neomer data.

### Patients page (genomes/exomes)

The Patient Data page provides functionalities that enable users to filter patient records based on specific individual characteristics (Fig. 3C). Users can select particular patient records and download the selected entries in Comma-Separated Values (CSV) format. Additionally, the interface allows customization of visible columns, enhancing usability. Both genomic and exomic datasets within the patient data exhibit complete feature parity, ensuring uniformity. Selecting an individual patient directs the user to a detailed Patient page (Fig. 3D), which provides comprehensive information on patient characteristics, including the cancer type and associated organ. From this detailed view, users can perform a patient-specific neomer search by specifying a desired sequence length and inputting a text prefix. This functionality generates a list of the top matching neomers of the specified length that begin with the provided text. Moreover, upon selecting a neomer from the results of the prefix-based search, users can examine its occurrence across different patients, cancer types, and organs, thereby enabling deeper investigation into the distribution of specific neomers.

### Downloads page

The neomerDB dataset is openly available for download via Zenodo repositories, providing users with flexible access to curated, cancer-specific genomic information. Downloads can be parameterized through the neomerDB website based on neomer length. Users can also distinguish between genomic and exomic datasets and further refine their selection by downloading the dataset in its entirety, by individual cancer type, or by cancer organ system. All datasets were generated using the neomer extraction and common variant filtering algorithms and are provided in compressed CSV format.

### Analysis and visualizations pages

**Figure 4:**
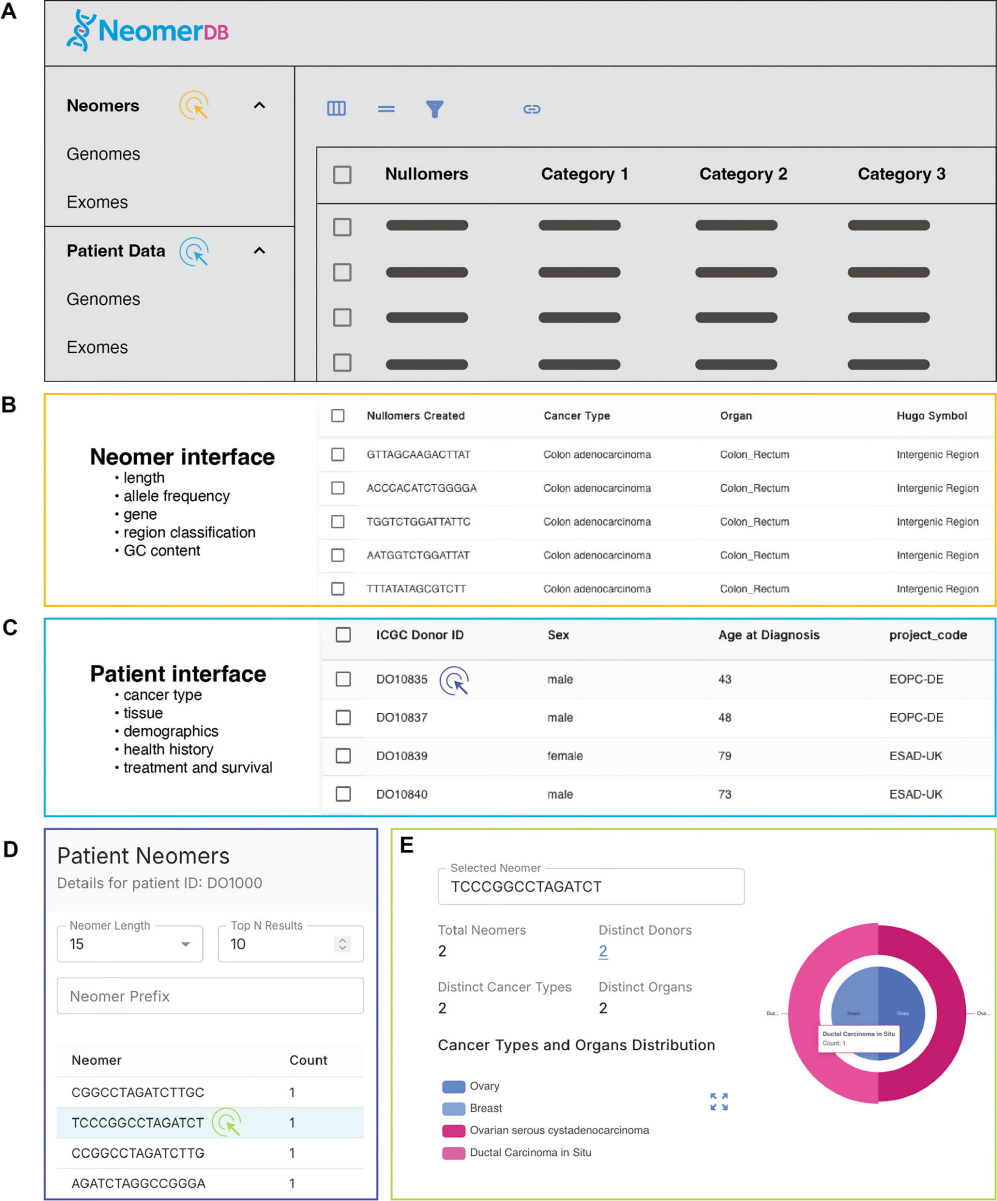
Interface of the visualizations offered by neomerDB. (A) Pie chart displaying the number of neomers identified per cancer type and (inset) across different *k*-mer lengths for a specific cancer type. (B) Heatmap displaying the Jaccard similarity index values representing the overlap between neomer sets across different cancer types. (C) Barplot displaying the number of neomers per donor.

NeomerDB provides multiple dynamic visualizations to explore the neomer data. These include pie charts that break down the number of neomers discovered per cancer type across *k*-mer lengths (Fig. 4A), and a breakdown of neomers discovered by the neomer length (Fig. 4A). To compare the proportion of neomers that are common between cancer types, the Jaccard index compares the shared neomers for every pair of cancer types (Fig. 4B). We find that the proportion of neomers shared between cancer types remains small in all cases, indicating the cancer-type specificity of neomer biomarkers. Finally, we provide a dynamic bar plot that is configurable per cancer type, cancer organ, and length and visualizes the number of neomers shared by exactly *N* patients (Fig. 4C). Together, these visualizations provide an intuitive and flexible interface for examining the specificity, prevalence, and distribution of neomers across various cancer types and patient cohorts.

### Case-study: detection of glioblastoma with neomers in cfDNA and cfRNA

We examined whether neomers detected in liquid biopsies could serve as cancer biomarkers through integrated analysis of cfDNA and cfRNA data. cfDNA and cfRNA were extracted from plasma samples collected from 29 glioblastoma patient samples and 9 non-cancer control samples. We used 15 bp neomers derived from two sources: glioblastoma patients’ genomes from PCAWG for cfDNA and exomes [17] for cfRNA analysis. We only included neomers present in at least two patients for both sources to ensure robustness.

**Figure 5:**
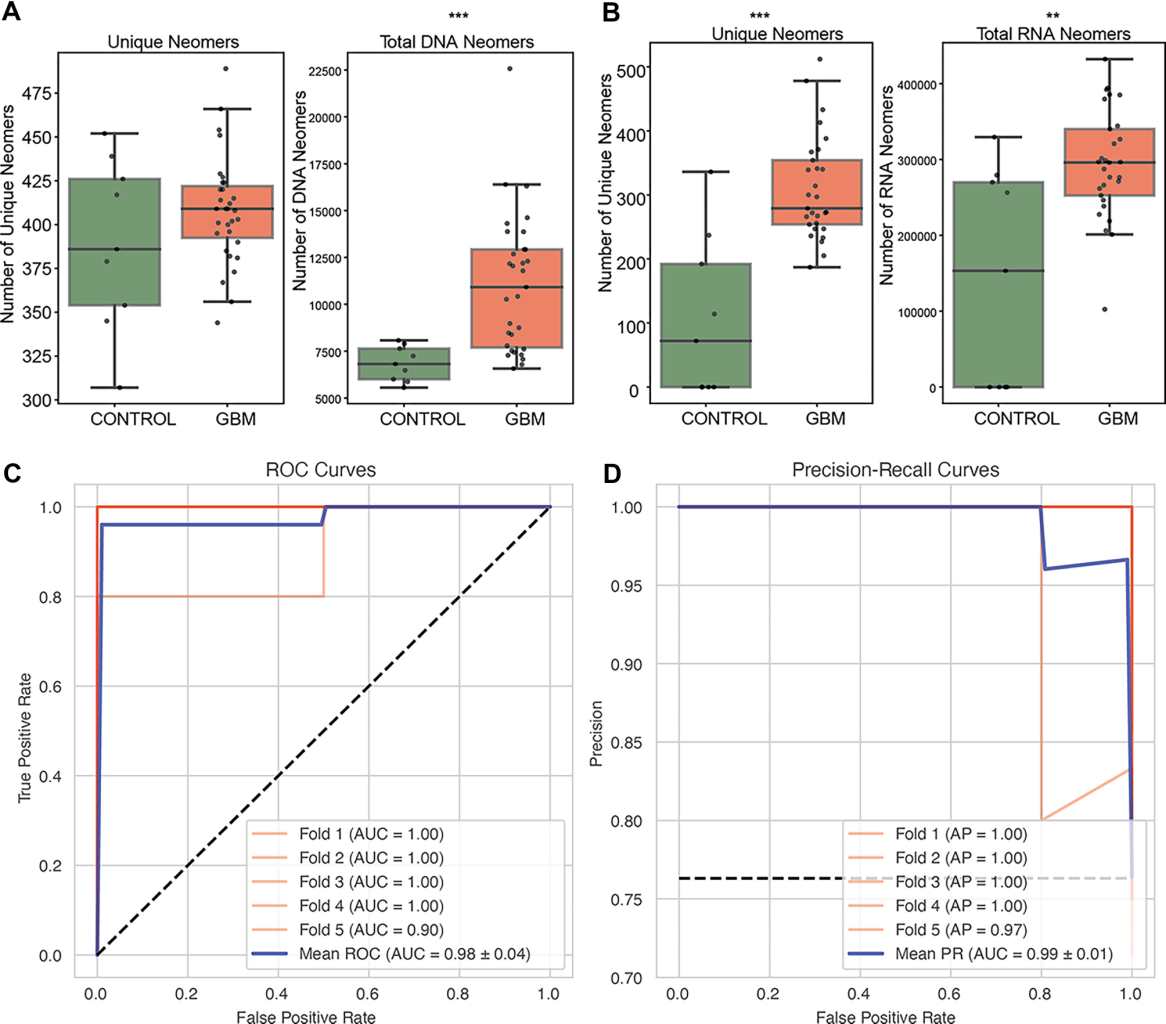
Performance of neomer biomarkers in cfDNA and cfRNA from liquid biopsies for glioblastoma detection. (A–B) Unique neomer counts and total neomer counts found in (A) cfDNA (Mann–whitney *U-*test, *P*-value = .27, .002) and (B) cfRNA (Mann–Whitney *U*-test, *P*-value = .002, .007). (C) ROC-AUC curves and (D) precision recall-AUC curves for glioblastoma across five-folds and averaged.

We first examined the number of unique neomers, and found that more exome neomers were found in glioblastoma (GBM) samples than in controls (DNA, 1.04×, Mann–Whitney *U*-test, *P*-value = .47; RNA, 1.59×, Mann–Whitney *U*-test, *P*-value = .002) (Fig. 5A). Next, we examined if the total counts of nullomers differed between controls and GBM samples (Fig. 5B). In both cfRNA and cfDNA, we found higher counts of nullomers in GBM samples (DNA, 1.59×, Mann–Whitney *U*-test, *P*-value = .0003; RNA, 2.60×, Mann–Whitney *U*-test, *P*-value = .0001). Next, we developed a multi-modal classifier that leveraged neomers in both cfDNA and cfRNA through a stacked ensemble model. We were able to detect GBM using 15 bp neomers with an average ROC-AUC score of 0.98 ± 0.04 (Fig. 5C) and a precision-recall score of 0.99 ± 0.01(Fig. 5D).

## Discussion

In this study, we present neomerDB, the first comprehensive database dedicated to neomer biomarkers across a wide range of cancer types and tissues. By systematically analysing over 12 000 tumour genomes and exomes, and cross-referencing these with more than 800 000 germline samples from diverse ancestries, we have constructed a high-confidence catalogue of neomers, *k*-mers that are absent from the healthy human genome but recurrently emerge in cancer, which can be leveraged for the development of cancer biomarkers. Our approach builds upon and significantly expands previous work on nullomers and neomers [8–14], providing a dynamic and user-friendly platform for identifying cancer-specific *k*-mer biomarkers. The integration of population-scale germline variant data represents a key innovation, enabling the exclusion of neomers arising from germline variants within or across populations, thereby enhancing the specificity of candidate biomarkers. Importantly, neomerDB allows for dynamic filtering based on recurrence across patients, cancer types, tumour stage, and likelihood of occurrence in different ancestries, offering a high degree of flexibility for both research and translational applications. These filters are complemented by dynamic tables and interactive visualizations that enable intuitive exploration of neomer data, enhancing interpretability and user engagement.

By leveraging neomers derived from both cfDNA and cfRNA, we were able to accurately detect glioblastoma, achieving high performance in both ROC-AUC and precision-recall metrics. This demonstrates that even in the context of low mutational burden and hard-to-detect tumours, neomers can serve as effective, sensitive, and specific biomarkers. Future work will extend this framework to additional cancer types to evaluate the generalizability and robustness of neomer-based detection. As sequencing technologies continue to improve and the costs of liquid biopsy-based testing decrease, we anticipate that neomer-based diagnostics can become a valuable tool in population surveillance, early detection, longitudinal monitoring, and minimal residual disease assessment of cancer patients. Neomers are likely to also be incorporated into multi-feature biomarker models to improve cancer care. Profiling neomers in liquid biopsies may also reveal tumour-specific vulnerabilities and inform on neoantigenic targets, offering opportunities to guide personalized therapeutic strategies.

Finally, we have made neomerDB freely available as a web-based platform with interactive visualizations, downloadable data, and comprehensive documentation. By lowering the barrier to access and exploration, we hope this resource will empower researchers, clinicians, and data scientists to explore neomers as a novel class of biomarkers, and catalyse further discovery in precision oncology.

## Supplementary Material

baag006_Supplemental_Files

## Data Availability

neomerDB dataset can be found in Zenodo with a stable version at https://zenodo.org/uploads/15518511 with DOI 10.5281/zenodo.15518511. neomerDB pipelines can be found on GitHub at this link https://github.com/Georgakopoulos-Soares-lab/neomers_pipeline neomerDB can be found on GitHub at the following links: front-end: https://github.com/Georgakopoulos-Soares-lab/neomerdb-ui; back-end: https://github.com/Georgakopoulos-Soares-lab/neomer_db_back.
