# Correction to: neomerDB: a comprehensive database of neomer biomarkers in cancer

**DOI:** 10.1093/database/baag043

**Published:** 2026-07-21

This is a correction to: Kimonas Provatas, Candace S Y Chan, Ioannis Kerasiotis, Eleftherios Bochalis, Akshatha Nayak, Brad E Zacharia, Georgios A Pavlopoulos, Wei Li, Ilias Georgakopoulos-Soares, neomerDB: a comprehensive database of neomer biomarkers in cancer, *Database*, Volume 2026, 2026, baag006, https://doi.org/10.1093/database/baag006

In the originally published version of this manuscript, a conflict of interest statement was omitted.

This has been corrected to read: “The authors declare the following competing interests: I.G.S., has previously filed patent applications covering embodiments and concepts on neomers” instead of: “None declared”.

