# Supplementary figures and images for: neomerDB: a comprehensive database of neomer biomarkers in cancer
Source: Database (Oxford). 2026 Feb 12;2026:baag006. doi: 10.1093/database/baag006 (PMC12895195; doi:10.1093/database/baag006)

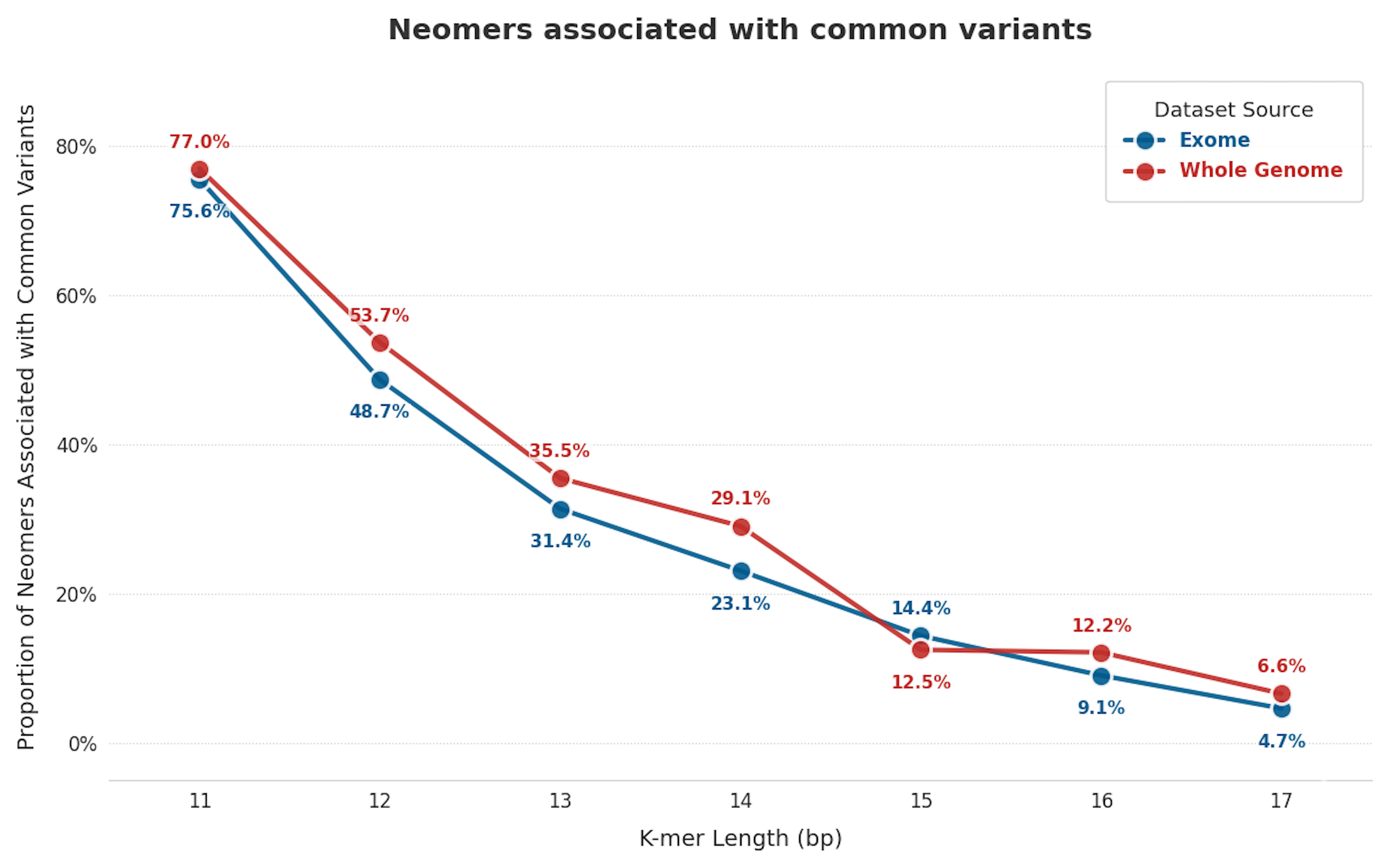

Supplement: baag006_Supplemental_Files [file baag006_supplemental_files.zip › Generated_Image_December_15,_2025_-_3_46PM.png]

Genome Neomers

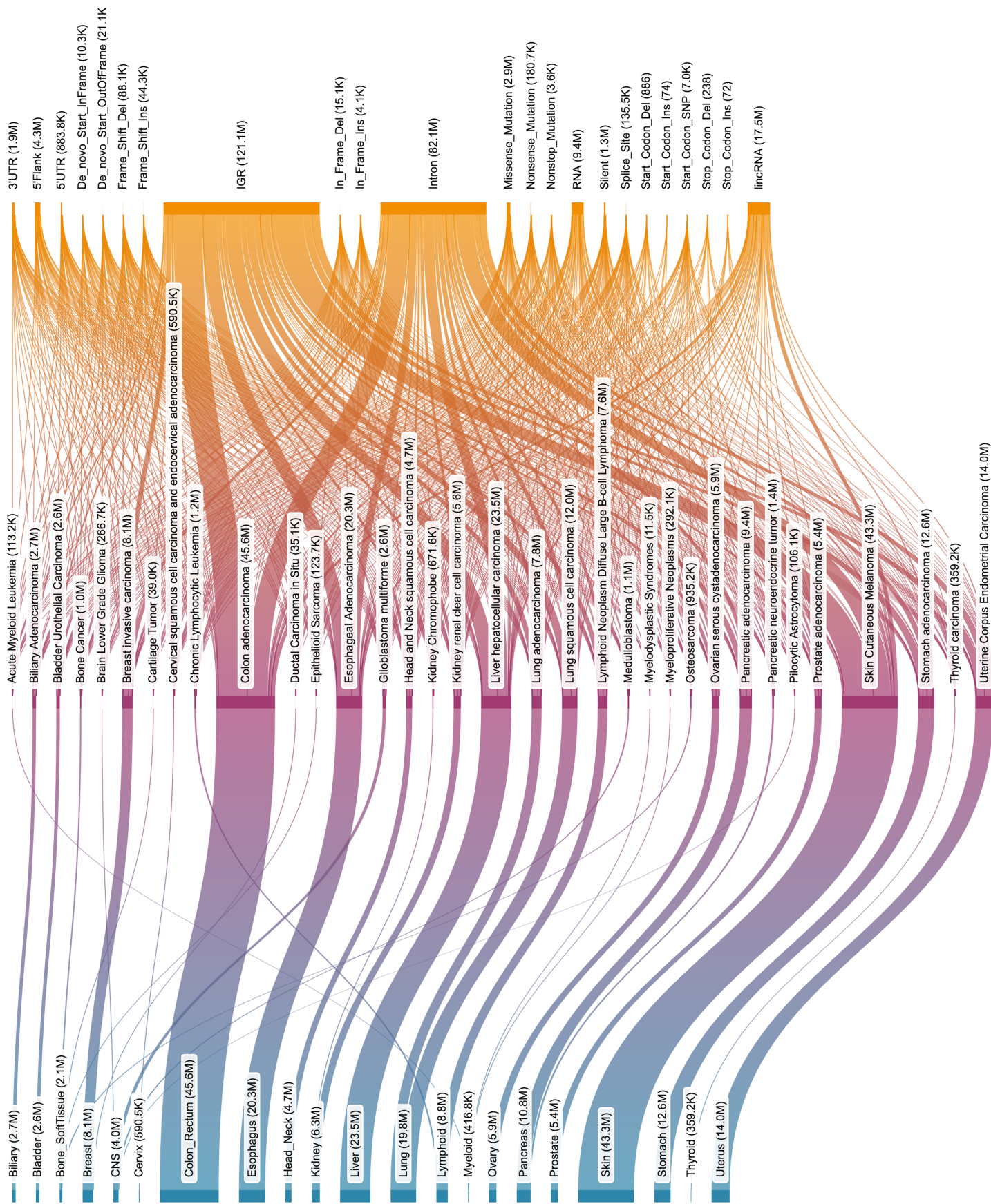

Supplement: baag006_Supplemental_Files [file baag006_supplemental_files.zip › genome.pdf]
